# The IRP/IRE system *in vivo*: insights from mouse models

**DOI:** 10.3389/fphar.2014.00176

**Published:** 2014-07-28

**Authors:** Nicole Wilkinson, Kostas Pantopoulos

**Affiliations:** Lady Davis Institute for Medical Research, Jewish General Hospital, and Department of Medicine, McGill UniversityMontreal, QC, Canada

**Keywords:** iron metabolism, ferritin, ferroportin, aconitase, transferrin receptor, HIF2α, DMT1, hepcidin

## Abstract

Iron regulatory proteins 1 and 2 (IRP1 and IRP2) post-transcriptionally control the expression of several mRNAs encoding proteins of iron, oxygen and energy metabolism. The mechanism involves their binding to iron responsive elements (IREs) in the untranslated regions of target mRNAs, thereby controlling mRNA translation or stability. Whereas IRP2 functions solely as an RNA-binding protein, IRP1 operates as either an RNA-binding protein or a cytosolic aconitase. Early experiments in cultured cells established a crucial role of IRPs in regulation of cellular iron metabolism. More recently, studies in mouse models with global or localized Irp1 and/or Irp2 deficiencies uncovered new physiological functions of IRPs in the context of systemic iron homeostasis. Thus, IRP1 emerged as a key regulator of erythropoiesis and iron absorption by controlling hypoxia inducible factor 2α (HIF2α) mRNA translation, while IRP2 appears to dominate the control of iron uptake and heme biosynthesis in erythroid progenitor cells by regulating the expression of transferrin receptor 1 (TfR1) and 5-aminolevulinic acid synthase 2 (ALAS2) mRNAs, respectively. Targeted disruption of either Irp1 or Irp2 in mice is associated with distinct phenotypic abnormalities. Thus, Irp1^−/−^ mice develop polycythemia and pulmonary hypertension, while Irp2^−/−^ mice present with microcytic anemia, iron overload in the intestine and the liver, and neurologic defects. Combined disruption of both Irp1 and Irp2 is incombatible with life and leads to early embryonic lethality. Mice with intestinal- or liver-specific disruption of both Irps are viable at birth but die later on due to malabsorption or liver failure, respectively. Adult mice lacking both Irps in the intestine exhibit a profound defect in dietary iron absorption due to a “mucosal block” that is caused by the de-repression of ferritin mRNA translation. Herein, we discuss the physiological function of the IRE/IRP regulatory system.

## Principles of mammalian iron metabolism

Iron is a vital micronutrient and constituent of several metalloproteins (Aisen et al., 2001). Even though it may acquire many oxidation states (from −2 to +6), within cells iron commonly alternates between the *ferrous* Fe(II) and *ferric* Fe(III) forms. Because of its abilities to coordinate with proteins and to act as both an electron donor and an electron acceptor, iron has been utilized throughout evolution by almost all living cells and organisms for metabolic purposes. Thus, iron is an integral component in: oxygen transport, through the heme moiety of hemoglobin; cellular respiration, as part of heme-containing cytochromes and Fe-S cluster-containing proteins of the *electron transport chain* (ETC); DNA synthesis and cellular growth, as part of the M2 subunit of ribonucleotide reductase.

As essential as iron is to survival, it can also be toxic. “Free” iron engages in Fenton chemistry to catalyze the production of hydroxyl radicals from superoxide and hydrogen peroxide (Papanikolaou and Pantopoulos, 2005). These are extremely reactive species that can damage lipid membranes, proteins and nucleic acids within a cell. To avoid the deleterious effects of the Fenton reaction, iron has to be shielded. Circulating iron is oxidized to Fe(III) and tightly binds to transferrin (Tf), which maintains it in a redox-inert state and delivers it to tissues (Gkouvatsos et al., 2012). Cellular iron uptake involves the binding of iron-loaded Tf to transferrin receptor 1 (TfR1) and the internalization of the complex by endocytosis (Aisen, 2004). Within endosomes, Fe(III) is released from Tf following acidification, and undergoes reduction to Fe(II). Subsequently, Fe(II) crosses the endosomal membrane via the divalent metal transporter 1 (DMT1). Internalized iron is mostly utilized in mitochondria for synthesis of heme and Fe-S clusters, and in the cytosol for incorporation into metalloproteins, while excess of iron is stored and detoxified within ferritin (Arosio et al., 2009). Ferritin is ubiquitously expressed in the cytosol of cells. It is comprised of 24 H- and L-polypeptide subunits that form a shell-like nanocage for iron storage, with a diameter of 7–8 nm. This can accommodate up to 4500 iron atoms, which are safely stored in the form of ferric oxy-hydroxide phosphate, following oxidation of Fe(II) to Fe(III) at the ferroxidase center of the H-ferritin subunit. In addition, a distinct ferritin isoform (M-ferritin) is expressed in mitochondria of some cell types, such as testicular Leydig cells, neuronal cells and pancreatic islets of Langherans (Levi and Arosio, 2004).

**Figure 1:**
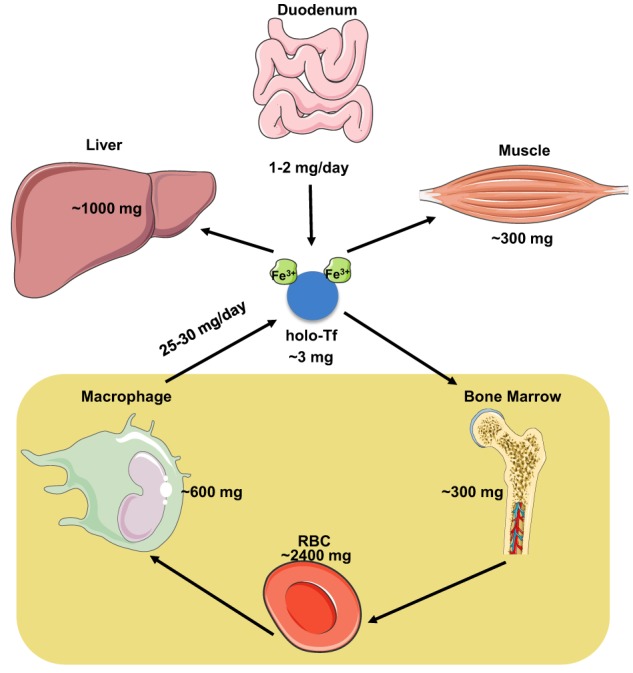
**Distribution and dynamics of iron traffic in the human body**.

The iron content of the adult human body ranges between 3 and 5 g (Gkouvatsos et al., 2012). Most of it (~70%) is utilized in hemoglobin of red blood cells (RBCs) in the bloodstream and of erythroid progenitor cells in the bone marrow, and is recycled by tissue macrophages at a rate of 25–30 mg/day (Figure 1). A substantial amount of body iron (up to 1 g) is stored within ferritin in the liver. Muscles contain ~300 mg of iron (mostly in myoglobin), and all other tissues (excluding the duodenum) merely ~8 mg. Circulating Tf-bound iron represents a small (~3 mg) but dynamic fraction that turns over ~10 times per day (Cavill, 2002). The Tf iron pool is primarily replenished by iron from senescent RBCs that is recycled via macrophages, and to a smaller extent by iron absorbed from the diet via duodenal enterocytes. In adults, dietary iron absorption (1–2 mg/day) serves to compensate for non-specific iron losses (by desquamation or bleeding).

**Figure 2:**
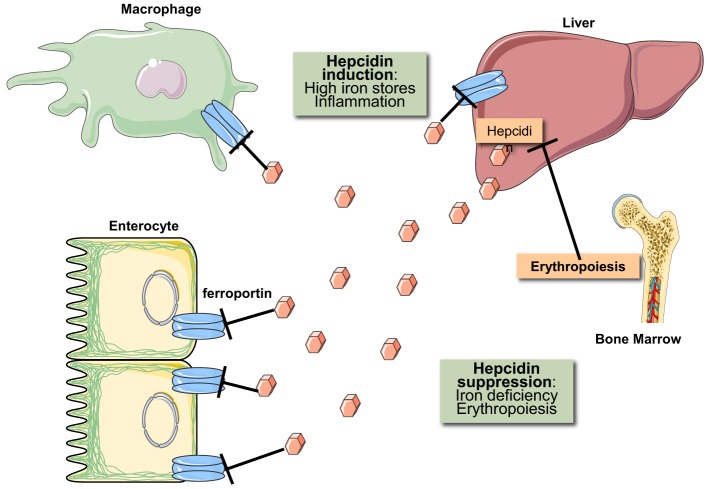
**Hormonal regulation of iron efflux from reticuloendothelial macrophages and duodenal enterocytes**. The iron regulatory hormone hepcidin is secreted by liver hepatocytes and targets the iron transporter ferroportin on the plasma membrane of iron-exporting cells. The binding of hepcidin promotes ferroportin degradation in lysosomes. Hepcidin expression is induced in response to high iron stores and inflammatory signals. Conversely, hepcidin expression is suppressed in response to iron deficiency and increased erythropoietic drive.

Iron metabolism is regulated at both the systemic and cellular levels. Systemic iron homeostasis is controlled through hepcidin, a liver-derived peptide hormone (Ganz, 2013). Iron and other stimuli (inflammation, endoplasmic reticulum stress) positively regulate hepcidin transcription, while erythropoietic drive suppresses it. Hepcidin binds to the cellular iron exporter ferroportin expressed on enterocytes, macrophages, and hepatocytes causing ferroportin's subsequent internalization and degradation (Nemeth et al., 2004). Ergo hepcidin functions to control iron absorption within the enterocytes and the efflux of iron into the bloodstream from macrophages and other iron exporting cells (Figure 2). Misregulation of hepcidin is associated with disease (hemochromatosis, when iron-regulation of hepcidin is blunted, and anemia, when hepcidin is induced by inflammatory pathways or by genetic inactivation of its inhibitor *TMPRSS6*) (Sebastiani and Pantopoulos, 2011). Cellular iron metabolism is controlled through the IRE/IRP system (Wang and Pantopoulos, 2011; Joshi et al., 2012), which is the focus of this review.

## Coordinate post-transcriptional regulation of cellular iron metabolism via IRE/IRP interactions

**Figure 3:**
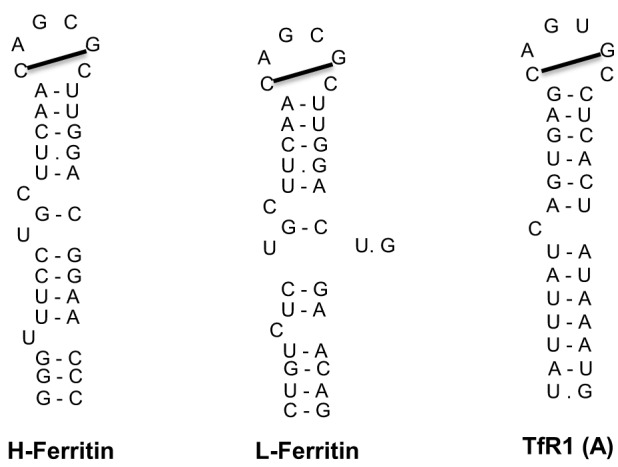
**IRE motifs in the untranslated regions of the mRNAs encoding human H-ferritin, L-ferritin and TfR1 (only the A IRE of TfR1 mRNA is shown)**. Watson-Crick base pairing is depicted by a dash and non-Watson-Crick interactions by a dot. The line connecting the 1st and 5th nucleotide of the IRE loop indicates Watson-Crick base pairing.

The IRE/IRP regulatory system was first described in the late 1980s with the discovery of iron responsive elements (IREs) in the untranslated regions (UTRs) of the mRNAs encoding ferritin (both H- and L-subunits) and TfR1 (Hentze et al., 1987; Casey et al., 1988; Müllner and Kühn, 1988); see Figure 3. IREs are highly conserved hairpin structures of 25–30 nucleotides (Piccinelli and Samuelsson, 2007). They contain a stem that is stabilized by base pairing, and a loop with the sequence 5′-CAGUGH-3′ (H denotes A, C or U). The stem is interrupted to an upper and lower part by an unpaired C residue or an asymmetric UGC/C bulge/loop. H- and L-ferritin mRNAs contain a single IRE in their 5′ UTR, which is located relatively close to the cap structure at the 5′ end. TfR1 mRNA contains five IREs in its 3′ UTR.

**Figure 4:**
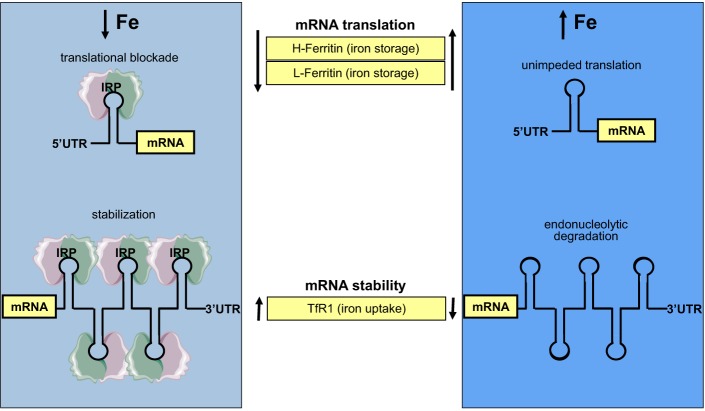
**Coordinate iron-dependent regulation of ferritin and TfR1 mRNA expression by IRE/IRP interactions**. Iron deficiency promotes the binding of IRPs to cognate IREs in the untranslated regions of ferritin and TfR1 mRNAs. These inhibit ferritin mRNA translation and stabilize TfR1 mRNA against endonucleolytic degradation. In iron-replete cells, IRPs do not bind to IREs, allowing ferritin mRNA translation and TfR1 mRNA degradation.

The IREs constitute binding sites of two cytoplasmic iron regulatory proteins, IRP1 and IRP2 (Rouault, 2006). Under conditions of iron deficiency, IRP1 and IRP2 bind with high affinity to the IRE in H- and L-ferritin mRNAs, and thereby inhibit their translation by a steric hindrance mechanism. Likewise, they bind to the IREs in TfR1 mRNA and thereby protect it against endonucleolytic degradation. This homeostatic response mediates increased cellular iron uptake from Tf and prevents storage of the metal. By contrast, in iron-replete cells the IRE-binding activities of IRP1 and IRP2 are diminished, allowing TfR1 mRNA degradation and ferritin mRNA translation. This inhibits further iron uptake and stimulates storage of excessive intracellular iron within ferritin. A scheme with the coordinate post-transcriptional regulation of ferritin and TfR1 by IRE/IRP interactions is shown in Figure 4.

**Figure 5:**
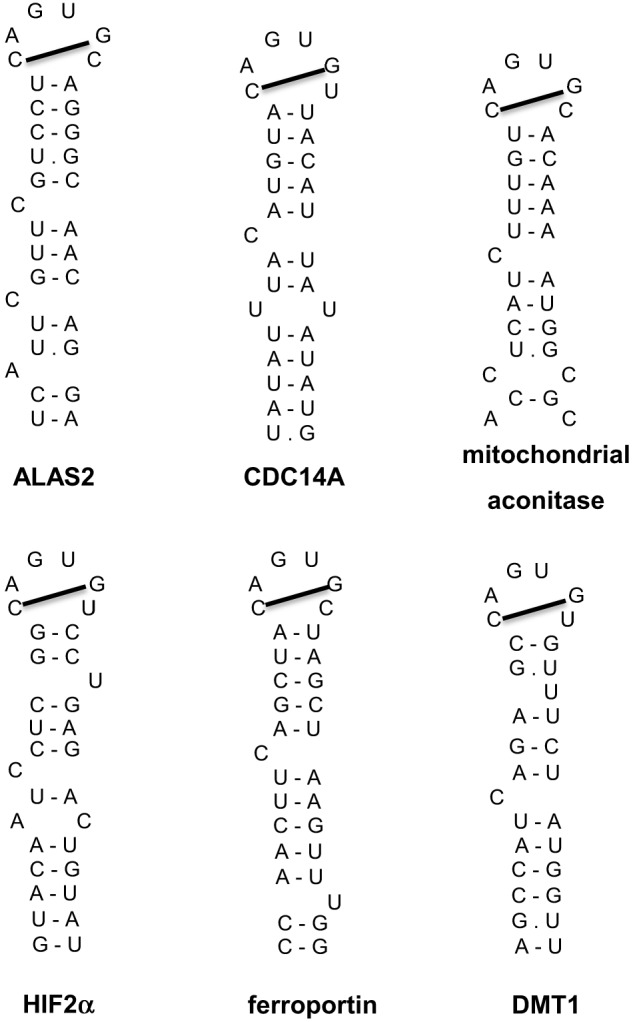
**IRE motifs in the untranslated regions of the mRNAs encoding human ALAS2, CDC14A, mitochondrial aconitase, HIF2α, ferroportin, and DMT1**. Watson-Crick base pairing is depicted by a dash and non-Watson-Crick interactions by a dot. The line connecting the 1st and 5th nucleotide of the IRE loop indicates Watson-Crick base pairing.

Additional IRE-containing mRNAs (Figure 5) have been discovered by computational and biochemical approaches, as well as high throughput screens (Dandekar and Hentze, 1995; Gunshin et al., 1997; McKie et al., 2000; Sanchez et al., 2006, 2007, 2011). These include the mRNAs encoding the iron transporters DMT1 and ferroportin, the enzyme 5-aminolevulinic acid synthase 2 (ALAS2) that catalyzes the first reaction for heme biosynthesis in erythroid progenitor cells, the enzyme of the citric acid cycle mitochondrial aconitase, the cell cycle regulator CDC14A, and hypoxia inducible factor 2 alpha (HIF2α), a transcription factor that orchestrates molecular responses to hypoxia. All these transcripts harbor a single IRE. The IRE of ferroportin, ALAS2, mitochondrial aconitase and HIF2α mRNAs is localized in the 5′ UTR and functions as a translational control element, analogous to ferritin IRE. The IRE of DMT1 and CDC14A mRNAs is localized in the 3′ UTR and appears to function as an mRNA stability element. Post-transcriptional regulation of DMT1 expression by the IRE/IRP system is cell-specific (Gunshin et al., 2001) and depends, at least partially, on an alternatively transcribed upstream exon at the 5′ end of DMT1 mRNA (Hubert and Hentze, 2002). Notably, earlier experiments showed that the presence of at least three 3′ UTR IREs is essential for iron-dependent regulation of TfR1 mRNA (Casey et al., 1989), which remains the only transcript containing multiple IREs. It should also be noted that DMT1, ferroportin and CDC14A mRNAs include additional non-IRE-containing isoforms, which are generated by alternative splicing and exhibit differential tissue distribution (Hubert and Hentze, 2002; Sanchez et al., 2006; Zhang et al., 2009).

Another level of complexity is added by the fact that iron regulation of HIF2α, ferroportin and DMT1 integrates multiple and often opposing signals (Mastrogiannaki et al., 2013; Shah and Xie, 2014). Thus, while iron stimulates HIF2α and ferroportin mRNA translation, it also promotes the degradation of HIF2α via iron- and oxygen-dependent prolyl hydroxylases (PHDs) and the von Hippel Lindau E3 ubiquitin ligase (pVHL) (Schofield and Ratcliffe, 2004), and the degradation of ferroportin via hepcidin (Ganz, 2013). Conversely, iron deficiency inhibits translation of HIF2α and ferroportin mRNAs but promotes stabilization of the proteins. On the other hand, iron deficiency leads to HIF2α-mediated transcriptional activation of duodenal DMT1 and ferroportin, the apical and basolateral transporters of iron in enterocytes (see below).

## Sensing of intracellular iron by IRP1 and IRP2

IRP1 and IRP2 are homologous to mitochondrial aconitase (Rouault, 2006; Wang and Pantopoulos, 2011). A characteristic feature of this enzyme is the presence of a 4Fe-4S cluster within its active site, which is indispensable for catalytic isomerization of citrate to iso-citrate. IRP1 assembles an aconitase-type 4Fe-4S cluster in response to increased intracellular iron levels. The complex pathway involves several co-factors, such as Nfs1 (ISCS), frataxin, ISCU, glutaredoxin 5 (*GLRX5*) and others. Assembly of the 4Fe-4S cluster alters the conformation of IRP1 and precludes IRE-binding (Dupuy et al., 2006; Walden et al., 2006). At the same time holo-IRP1 (containing the 4Fe-4S cluster) acquires enzymatic activity as cytosolic aconitase, which is comparable to that of its mitochondrial counterpart. Iron starvation, as well as other signals, such as nitric oxide (NO) or hydrogen peroxide (H_2_O_2_), promotes the loss of the 4Fe-4S cluster and conversion of apo-IRP1 to an IRE-binding protein (Haile et al., 1992; Drapier et al., 1993; Weiss et al., 1993; Pantopoulos and Hentze, 1995). Thus, IRP1 is a bifunctional protein that is regulated by an unusual 4Fe-4S cluster switch. Holo-IRP1 is stabilized by hypoxia and at oxygen concentrations ranging between 3 and 6%, which reflect physiological tissue oxygenation (Meyron-Holtz et al., 2004b). Consistently with this notion, IRP1 predominates in the cytosolic aconitase form within tissues, but a fraction of apo-IRP1 is readily detectable by virtue of its IRE-binding activity (Meyron-Holtz et al., 2004a).

Contrary to IRP1, IRP2 does not bind an iron-sulfur cluster and is regulated at the level of protein stability. IRP2 accumulates in iron-starved and/or hypoxic cells, and undergoes proteasomal degradation in iron-replete oxygenated cells. Mechanistically, this involves ubiquitination of IRP2 by an E3 ubiquitin ligase complex consisting of the F-box protein FBXL5 and the auxiliary proteins SKP1, CUL1, and RBX1 (Salahudeen et al., 2009; Vashisht et al., 2009). FBXL5 exhibits genuine iron-sensing properties, as it is stabilized in the presence of iron and oxygen by forming a Fe-O-Fe center within its N-terminal hemerythrin-like domain. Loss of this center in iron-starved and/or hypoxic cells exposes a degron via a conformational change (Chollangi et al., 2012; Thompson et al., 2012). This allows proteasomal degradation of FBXL5, which leads to concomitant accumulation of IRP2. Interestingly, FBXL5 can also promote the ubiquitination and degradation of IRP1 mutants that cannot form a 4Fe-4S cluster (Salahudeen et al., 2009; Vashisht et al., 2009). Proteasomal degradation of wild type apo-IRP1 under conditions of impairment of the iron-sulfur cluster assembly pathway has been proposed to operate as a reserve mechanism to control the IRE-binding activity of IRP1 (Clarke et al., 2006; Wang et al., 2007).

## Human diseases linked to the IRE/IRP system

Mutations in the IRE of L-ferritin mRNA that abrogate IRP binding are causatively linked to the hereditary hyperferritinemia-cataract syndrome (HHCS) (Beaumont et al., 1995). The hallmark of this disorder is overexpression of serum ferritin (up to 20-fold) in the absence of systemic iron overload or inflammation (Yin et al., 2014). Patients also have an increased tendency to develop bilateral cataract, which is possibly caused by accumulation of non-functional L-ferritin homopolymers in the lens (Levi et al., 1998). HHCS is transmitted in an autosomal dominant manner. Several HHCS-associated mutations in L-ferritin IRE have been described (Luscieti et al., 2013). Mutations affecting the loop structure or the C bulge result in the highest levels of serum ferritin and increased severity of cataract when compared with mutations in the stem structure of the IRE (Cazzola et al., 1997). Overall, the severity of the clinical phenotype appears to correlate with the degree of inhibition in IRP binding (Allerson et al., 1999). Nevertheless, the involvement of additional factors (genetic, environmental, inflammatory) has not been excluded (Roetto et al., 2002).

A point mutation (A49U) in the loop of H-ferritin IRE has been associated with an autosomal dominant iron overload disorder in a Japanese family (Kato et al., 2001). Iron deposits were documented primarily in hepatocytes, but also in some Kupffer cells. The A49U mutation increased the affinity of IRPs for H-ferritin IRE and suppressed H-ferritin expression in cultured cells (Kato et al., 2001). Nevertheless, it remains unclear how this response promotes iron overload. The lack of follow-up studies to support the validity of these findings should also be noted.

Disruption of the zebrafish *glrx5* gene, encoding glutaredoxin 5, leads to constitutive activation of IRP1 for IRE-binding, due to defective assembly of its 4Fe-4S cluster (Wingert et al., 2005). In a case report, a patient with *GLRX5* deficiency developed a sideroblastic-like anemia with microcytosis and systemic iron overload (Camaschella et al., 2007). This disease is caused by IRP1-mediated suppression of ALAS2 mRNA translation, which results in impaired heme biosynthesis. A secondary effect is the depletion of cytosolic iron and the development of mitochondrial iron overload (Ye et al., 2010).

The above examples represent the only documented human disorders that are causatively linked to defects in the IRE/IRP system (Table 1). Genome-wide association studies (GWAS) showed that the IRP2 gene (*IREB2*) confers susceptibility to chronic obstructive pulmonary disease (COPD) (Demeo et al., 2009; Chappell et al., 2011; Zhou et al., 2012) and lies within a lung cancer-associated locus (Hansen et al., 2010; Cho et al., 2012; Fehringer et al., 2012). Apart from the identification of single nucleotide polymorphisms (SNPs) in *IREB2*, it was shown that COPD patients exhibit increased IRP2 mRNA and protein expression (Demeo et al., 2009) and it was speculated that this may contribute to iron accumulation in the lungs. *IREB2* polymorphisms have also been associated with Alzheimer's disease (Coon et al., 2006). Interestingly, biochemical studies showed that IRP1 selectively regulates translation of amyloid precursor protein (APP) mRNA by binding to an atypical putative IRE motif (Cho et al., 2010), which may provide another connection of the IRE/IRP system with Alzheimer's disease. The IRP1 gene (*ACO1*) has been associated with cutaneous malignant melanoma (Yang et al., 2010) and neuropathic pain in HIV-infected patients (Kallianpur et al., 2014), while the IRE-binding activity of IRP1 was reported to be increased in Friedreichs' ataxia (Lobmayr et al., 2005) and in Parkinson's disease (Faucheux et al., 2002). Finally, polymorphisms in both *ACO1* and *IREB2* have been linked to age-related macular degeneration (Synowiec et al., 2012). GWAS data with *ACO1* and *IREB2* are summarized in Table 2. The molecular mechanisms by which IRPs may contribute to pathogenesis of the above disorders remain to be established.

**Table 1:** **Human disorders that are causatively linked to defects in the IRE/IRP system**.

**Human disease**	**Mutation**	**Phenotype**	**References**
Hereditary hyperferritinemia-cataract syndrome (HHCS)	Mutations in the IRE of L-ferritin mRNA that impair IRP binding	Overexpression of serum ferritin in the absence of systemic iron overload or inflammation. Tendency for the development of bilateral cataract	(Beaumont et al., 1995; other papers identified further mutations)
Iron overload disorder with autosomal dominant transmission	Point mutation in the IRE loop of H-ferritin that increases IRP binding	Suppression of H-ferritin leading to an iron overload disorder phenotypically related to hemochromatosis	Kato et al., 2001
Sideroblastic-like anemia with iron overload	*GLRX5* deficiency leading to increased IRE-binding activity of IRP1 and suppression of ALAS2 mRNA translation	Development of a sideroblastic-like anemia with microcytosis and systemic iron overload	Camaschella et al., 2007

**Table 2:** **Genome-wide association studies (GWAS) with the IRPI and IRP2 genes, *ACO1* and *IREB2*, respectively**.

**Gene**	**Disease**	**Association**	**References**
*IREB2*	Chronic obstructive pulmonary disease (COPD)	Patients with COPD have increased IRP2 mRNA and protein expression in addition to a single nucleotide polymorphism in *IREB2*	Demeo et al., 2009; Chappell et al., 2011; Zhou et al., 2012
*IREB2*	Lung cancer	Lies within a cancer-associated locus	Hansen et al., 2010; Cho et al., 2012; Fehringer et al., 2012
*IREB2*	Alzheimer's disease	*IREB2* polymorphism have been associated with Alzheimer's disease	Coon et al., 2006
*ACO1*	Neuropathic pain in HIV-infected patients	*ACO1* was significantly associated with distal neuropathic pain and pain severity in HIV-infected patients on antiretroviral therapy	Kallianpur et al., 2014
*ACO1*	Cutaneous malignant melanoma(CMM)	*ACO1* was significantly associated with CMM and training ability	Yang et al., 2010
*ACO1* and *IREB2*	Macular degeneration	Polymorphisms in both *ACO1* and *IREB2* have been linked to age related macular degeneration	Synowiec et al., 2012

## Mouse models with ubiquitous aberration in the IRE/IRP system

Mice with ubiquitous ablation of both Irp1 and Irp2 cannot be generated because the embryos do not survive the blastocyst stage, possibly due to misregulation of iron metabolism (Smith et al., 2006). These data highlight the physiological significance of the IRE/IRP system in early development. On the other hand, single Irp1^−/−^ or Irp2^−/−^ mice are viable. At first glance, this suggests that Irp1 and Irp2 share overlapping functions *in vivo*. Nonetheless, Irp1^−/−^ and Irp2^−/−^ mice manifest distinct phenotypes, indicating variable target specificity. The phenotypic features of current mouse models with global or tissue-specific “loss” or “gain” of Irp1 and/or Irp2 functions are summarized in Table 3.

**Table 3:** **Phenotypic features of mouse models with global or tissue-specific “loss” or “gain” of Irp1 and/or Irp2 functions**.

**Mouse model**	**Site of modification**	**Generation credit**	**Phenotype**	**Phenotype credit**
Irp1^−/−^	Global	Meyron-Holtz et al., 2004a;Galy et al., 2004	Polycythemia, stress erythropoiesis, splenomegaly, and increased expression of erythropoietin	Anderson et al., 2013; Ghosh et al., 2013; Wilkinson and Pantopoulos, 2013
			Pulmonary hypertension, cardiac hypertrophy and cardiac fibrosis. Mice succumb to hemorrhages when fed an iron-deficient diet	Ghosh et al., 2013
			Increased expression of Dmt1, ferroportin and Dcytb mRNAs in the duodenum	Anderson et al., 2013
			Misregulation ferritin and Tfr1 expression in the kidney and brown fat	Meyron-Holtz et al., 2004a
			Efficient inflammatory signaling response to turpentine	Viatte et al., 2009
			Increased ferroportin expression on splenic macrophages and decreased hepcidin mRNA levels in the liver	Wilkinson and Pantopoulos, 2013
Irp2^−/−^	Global	LaVaute et al., 2001; Galy et al., 2004; Zumbrennen-Bullough et al., 2014	Microcytic hypochromic anemia with mild duodenal and hepatic iron overload and splenic iron deficiency	Cooperman et al., 2005; Galy et al., 2005b
			Reduced TfR1 expression in erythroid precursors	Cooperman et al., 2005; Galy et al., 2005b
			High levels of protoporphyrin IX in erythroid precursors	Cooperman et al., 2005
			Increased ferritin levels in all tissues	Cooperman et al., 2005
			Iron overload in neurons and progressive neurodegeneration	LaVaute et al., 2001; Ghosh et al., 2006
			Efficient inflammatory signaling response to turpentine	Viatte et al., 2009
			Mild neurological and behavioral defects, as well as nociception	Zumbrennen-Bullough et al., 2014
			Minor performance deficits in specific neurological tests (motor coordination and balance)	Galy et al., 2006
Irp2^−/−^	Liver-specific	Ferring-Appel et al., 2009	Mild hepatic iron overload	Ferring-Appel et al., 2009
Irp2^−/−^	Intestinal-specific	Ferring-Appel et al., 2009	Mild duodenal iron overload	Ferring-Appel et al., 2009
Irp2^−/−^	Macrophage-specific	Ferring-Appel et al., 2009	No pathology	Ferring-Appel et al., 2009
Irp1^−/−^Irp2^−/−^	Global	Smith et al., 2006	Embryonic lethality at the blastocyst stage of development	Smith et al., 2006
Irp1^+/-^Irp2^−/−^	Global	Smith et al., 2004	More serve presentation of neuronal pathology than that of Irp2^−/−^ mice	Smith et al., 2004
			Neuronal pathology partially rescued by the pharmacological activation of endogenous Irp1	Ghosh et al., 2008
Irp1^−/−^Irp2^−/−^	Liver-specific	Galy et al., 2010	Early lethality within 1–2 weeks after birth due to liver failure	Galy et al., 2010
Irp1^−/−^Irp2^−/−^	Intestinal-specific	Galy et al., 2008	Growth retardation, early death (within 30 days) due to dehydration. Increased expression of ferritin, ferroportin, and Dmt1	Galy et al., 2008
Irp1^−/−^Irp2^−/−^	Adult ligand-induced intestinal-specific	Galy et al., 2013	Increased expression of ferritin leading to “mucosal block,” in spite of increased expression of ferroportin and Dmt1	Galy et al., 2013
Irp1 “gain of function” due to expression of a constitutive IRP1 transgene	Global	Casarrubea et al., 2013	Macrocytic erythropenia due to impaired erythroid differentiation	Casarrubea et al., 2013
Irp2 “gain of function” due to disruption of Fbxl5	Global	Moroishi et al., 2011; Ruiz et al., 2013	Embryonic lethality	Moroishi et al., 2011; Ruiz et al., 2013
Irp2 “gain of function” due to disruption of Fbxl5	Liver-specific	Moroishi et al., 2011	Hepatic iron overload and steatohepatitis; low hepcidin mRNA levels. Mice succumb to lethal liver failure when fed a high-iron diet	Moroishi et al., 2011; Ruiz et al., 2013

Irp1^−/−^ mice were initially reported to lack any overt abnormalities (Meyron-Holtz et al., 2004a; Galy et al., 2005a). Because they merely misregulated ferritin and TfR1 expression in the kidney and the brown fat, tissues with the highest abundance of IRP1, it was proposed that Irp2 dominates cellular iron metabolism in tissues, with Irp1 having an auxiliary physiological function (Meyron-Holtz et al., 2004a). In addition, Irp1^−/−^ (and Irp2^−/−^) mice did not exhibit any defects when challenged with turpentine to induce an inflammatory response (Viatte et al., 2009), despite the fact that IRPs are modulated in cultured cells by inflammatory reactive species such as H_2_O_2_ and NO (Weiss et al., 1993; Pantopoulos and Hentze, 1995; Wang et al., 2005; Hausmann et al., 2011).

Recently, Irp1^−/−^ mice were documented to develop polycythemia and pathological iron metabolism due to stress erythropoiesis, as well as pulmonary hypertension and cardiac hypertrophy and fibrosis (Anderson et al., 2013; Ghosh et al., 2013; Wilkinson and Pantopoulos, 2013). These phenotypes are attributed to relief of translational suppression of Hif2α mRNA, which leads to transcriptional activation of the downstream Hif2α targets erythropoietin and endothelin-1. Thus, murine Irp1 operates as specific regulator of the Hif2α IRE, in agreement with previous *in vitro* data (Zimmer et al., 2008). Irp1^−/−^ mice exhibit high mortality when placed on an iron-deficient diet, which is known to stabilize Hif2α, due to abdominal hemorrhages (Ghosh et al., 2013). When Irp1^−/−^ mice are fed with a standard diet, polycythemia attenuates after the 10th week of age (Wilkinson and Pantopoulos, 2013), possibly due to enhanced Hif2α degradation by the pVHL pathway. This may explain why their pathology escaped attention in the past.

Irp2^−/−^ mice develop microcytic hypochromic anemia and erythropoietic protoporphyria, associated with relatively mild duodenal and hepatic iron overload and splenic iron deficiency (Cooperman et al., 2005; Galy et al., 2005b). They exhibit a low Tfr1 content and express high levels of protoporphyrin IX in erythroid precursor cells. A closer look on the role of Irp1 and Irp2 in erythropoiesis, duodenal iron absorption and systemic iron metabolism will be provided in the next sections.

Global Irp2^−/−^ deficiency has also been associated with progressive neurodegeneration, loss of Purkinje neurons and iron overload in white matter areas of the brain (LaVaute et al., 2001; Ghosh et al., 2006), with more severe presentation in Irp1 haploinsufficient Irp1^+/−^Irp2^−/−^ mice (Smith et al., 2004). The neuronal pathology of Irp2^−/−^ mice can be partially rescued by pharmacological activation of endogenous Irp1 for IRE-binding (Ghosh et al., 2008). Mechanistically, the pathology may be caused by functional iron deficiency in neuronal cells due to de-repression of ferritin mRNA translation, which could result in enhanced iron storage and reduced availability for metabolic purposes (Jeong et al., 2011). This scenario is reminiscent of neuroferritinopathy, a neurodegenerative disease caused by expression of mutant L-ferritin (Burn and Chinnery, 2006). Suppression of Tfr1 and enhancement of ferroportin expression in Irp2^−/−^ neurons could also contribute to pathology. Nevertheless, isogenic Irp2^−/−^ mice generated by another strategy did not develop any signs of neurodegeneration but rather manifested minor performance deficits in specific neurological tests (motor coordination and balance) (Galy et al., 2006). A third independent Irp2^−/−^ mouse strain was recently generated and analyzed, corroborating the abnormalities in erythropoiesis and brain iron metabolism of these animals; the latter were linked to mild neurological and behavioral defects, as well as nociception (Zumbrennen-Bullough et al., 2014). The discrepancies in the phenotypes of the three Irp2^−/−^ mouse lines may be related to the targeting strategies and their impact on flanking genomic sequences, genetic factors, and possibly also environmental factors.

Ubiquitous Irp1 “gain of function” mice were generated by the transgenic expression of a constitutively active IRP1 mutant from the Rosa26 locus (Casarrubea et al., 2013). Expression of the transgene was relatively low and the mice developed erythropoietic abnormalities (macrocytic erythropenia due to impaired erythroid differentiation).

The targeted disruption of Fbxl5 could yield ubiquitous Irp2 “gain of function” mice. Nevertheless, Fbxl5^−/−^ mice are not viable and die during embryogenesis (Moroishi et al., 2011; Ruiz et al., 2013). This phenotype is rescued by concomitant disruption of Irp2, but not Irp1, indicating that the major physiological function of Fbxl5 is to regulate Irp2.

## The IRE/IRP system in erythropoiesis

Most iron in the body is utilized in erythropoiesis, a process that occurs mainly in the bone marrow of humans and in the bone marrow and spleen of mice (Cavill, 2002). Erythroid precursor cells express high levels of TfR1 and consume the majority of Tf-bound iron for the production of heme, the oxygen-binding moiety of hemoglobin (Ponka, 1997). An adult human produces about 2 million RBCs per second. Increased erythropoietic drive is known to stimulate iron absorption (Finch, 1994). This is mediated by suppression of hepcidin expression, which allows increased iron efflux to the bloodstream from duodenal enterocytes and tissue macrophages (Ganz, 2013). During iron deficiency, erythroid precursor cells will have priority for iron utilization, for the production of RBCs, over cells in other tissues (Finch, 1994). Maturation of erythroid cell is induced by erythropoeitin (EPO), a circulating glycoprotein hormone that prevents apoptosis of erythroid progenitor cells (Koury and Bondurant, 1990). EPO is primarily synthesized in peritubular interstitial fibroblasts of the kidney, and to a smaller extent in hepatocytes of the liver. Murine Epo is transcriptionally activated during hypoxemia by Hif2α (Rankin et al., 2007; Kapitsinou et al., 2010), while Hif2α indirectly suppresses hepcidin expression by stimulating erythropoiesis via Epo (Liu et al., 2012; Mastrogiannaki et al., 2012). There are several points where the IRE/IRP system regulates erythropoiesis.

First, the phenotype of Irp1^−/−^ mice demonstrates that Irp1 operates as a key upstream regulator of Hif2α expression at the level of mRNA translation (Anderson et al., 2013; Ghosh et al., 2013; Wilkinson and Pantopoulos, 2013). Essentially, these animals exhibit features of Hif2α overexpression, which are also manifested in patients with Chuvash polycythemia (Ang et al., 2002), and other forms of familial polycythemia caused by “gain of function” HIF2α mutations (Percy et al., 2008), or inactivating mutations in pVHL, a negative regulator of HIF2α stability (Percy et al., 2006). One of them, is hyperproduction of Epo, which causes splenomegaly due to extramedullary erythropoiesis, promotes expansion of late stage basophilic erythroblasts, as well as polychromatic and orthochromatic erythroblasts, and finally leads to reticulocytosis and polycythemia. Importantly, overexpression of hepatic Epo is rescued by intercrossing Irp1^−/−^ and liver-specific Hif2α^−/−^mice (Wilkinson and Pantopoulos, 2013). On the other hand, the defects in erythroid differentiation documented in the constitutive IRP1 transgenic mice can be attributed to reduced Hif2α expression (Casarrubea et al., 2013). Hence, the translational regulation of HIF2α mRNA by IRP1 links iron metabolism with erythropoiesis via EPO. Physiologically, this response probably serves to contain EPO expression and subsequent stimulation of erythropoiesis under conditions of iron deficiency. In addition, it is tempting to speculate that stress activation of IRP1 (by H_2_O_2_ or NO) (Weiss et al., 1993; Pantopoulos and Hentze, 1995) may contribute to the impairment of EPO production that is frequently observed under chronic inflammatory conditions or in chronic kidney disease (Weiss and Goodnough, 2005), via suppression of HIF2α mRNA translation.

**Figure 6:**
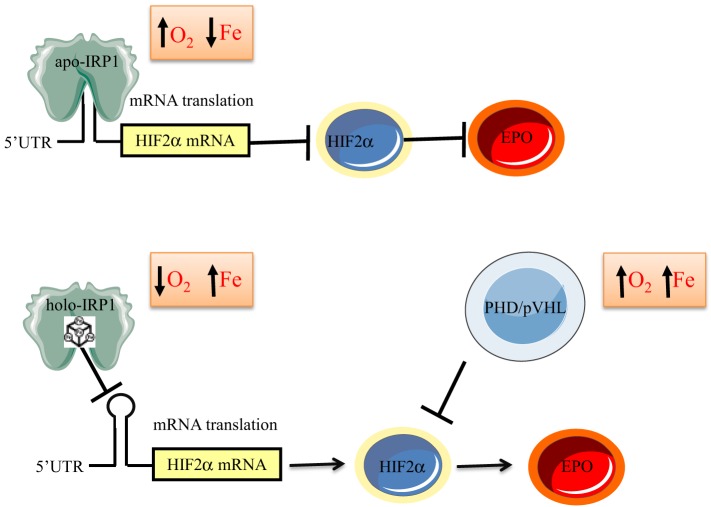
**Model for regulation of erythropoiesis by IRP1 via the HIF2α/EPO axis**. In iron-deficient or normoxic cells, apo-IRP1 limits HIF2α synthesis by binding to HIF2α mRNA. Iron-replete conditions favor conversion of apo- to holo-IRP1, at the expense of its IRE-binding activity, allowing HIF2α mRNA translation and transcriptional activation of its downstream target EPO. Hypoxia stabilizes holo-IRP1. Accumulation of HIF2α is antagonized by the PHD/pVHL degradation system, which is activated by oxygen and iron.

The above data suggest that IRP1 functions as an iron and oxygen sensor (Figure 6). According to this model, in normoxic renal interstitial fibroblasts the small steady-state fraction of apo-IRP1 inhibits HIF2α mRNA translation and thereby limits EPO production to physiological levels. This fraction is expected to increase in normoxic iron deficiency due to 4Fe-4S cluster disassembly, further suppressing HIF2α and EPO expression as a homeostatic response to reduced iron availability, consistently with iron-restricted erythropoiesis. Conversely, increased iron supply is expected to increase the abundance of holo-IRP1, allowing de-repression of HIF2α mRNA translation. This response is also favored by hypoxia, which is known to stabilize holo-IRP1. Thus, in hypoxic and/or iron-replete renal interstitial fibroblasts, unimpeded HIF2α mRNA translation leads to increased generation of EPO and thereby stimulates erythropoiesis, as a homeostatic adaptation to the scarcity of oxygen and/or the abundance of iron.

IRP2 is not involved in the regulation of HIF2α mRNA *in vivo*. This makes physiological sense, since IRP2 is highly active under hypoxia, when HIF2α synthesis is required. Nevertheless, the phenotype of Irp2^−/−^ mice suggests that IRP2 is a critical regulator of other erythropoietic pathways. These animals present with hypochromic microcytic anemia with reduced iron availability for erythropoiesis (Cooperman et al., 2005; Galy et al., 2005b; Zumbrennen-Bullough et al., 2014). This is not a result of global iron deficiency but rather misregulation of cellular iron traffic. Irp2^−/−^ erythroid cells exhibit a reduced Tfr1 content, apparently due to lack of Tfr1 mRNA stabilization in the absence of Irp2. As a result, these cells fail to acquire sufficient amounts of iron for erythropoiesis, in spite of the physiological iron supply, which is reflected in the normal Tf saturation and total iron-binding capacity (TIBC) in serum. Therefore, Irp2 appears to act as major activator of Tfr1 in erythroid cells. It is conceivable that this function is more pronounced at earlier stages of erythroid development, considering that during terminal erythroid differentiation, Tfr1 mRNA stability remains unresponsive to iron, bypassing the IRE/IRP checkpoint (Schranzhofer et al., 2006).

Irp2^−/−^ mice also manifest very high levels of free and zinc protoporphyrin IX in RBCs (~200-fold increase for free protoporphyrin IX), consistently with erythropoietic protoporphyria (Cooperman et al., 2005). This is very likely caused by enhanced heme biosynthetic activity due to de-repression of Alas2 mRNA translation. In the absence of adequate iron, it results in accumulation of free protoporphyrin IX and in incorporation of zinc in the protoporphyrin IX ring. Therefore, IRP2 also acts as a major regulator of ALAS2 mRNA translation via the IRE in its 5′ UTR. The interpretation that IRP2 regulates erythroid iron uptake and utilization by controlling expression of the IRE-containing TfR1 and ALAS2 mRNAs, respectively, is also supported by data with tissue-specific Irp2^−/−^ mice. Thus, liver- or intestinal-specific ablation of Irp2 recapitulates the iron overload phenotype of these tissues in ubiquitous Irp2^−/−^ mice, but fails to promote microcytic anemia (Ferring-Appel et al., 2009).

## The IRE/IRP system in dietary iron absorption

Dietary iron absorption takes place in brush border enterocytes of the duodenum, especially in the upper tract. The pathway is highly regulated at different levels. Hormonal regulation via hepcidin is well established. Hepcidin is produced in the liver in response to high serum or hepatic iron (Corradini et al., 2011; Ramos et al., 2011) and limits further iron absorption by promoting the degradation of duodenal ferroportin (Nemeth et al., 2004; Chung et al., 2009), the basolateral iron transporter. Hepcidin may also promote degradation of DMT1, the apical iron transporter, by the proteasome (Chung et al., 2009; Brasse-Lagnel et al., 2011). Both ferroportin (Taylor et al., 2011) and DMT1 (Mastrogiannaki et al., 2009; Shah et al., 2009) are transcriptionally activated in iron-deficient enterocytes by HIF2α. In addition, ferroportin and DMT1 could be directly regulated by IRPs at the levels of mRNA translation or stability, respectively. Duodenal enterocytes also express ferritin, which limits excessive iron transfer to the circulation (Vanoaica et al., 2010). TfR1 expression is restricted to non-absorptive enterocytes in the intestinal crypts (Waheed et al., 1999).

Iron deficiency elicits multiple and antagonistic signals on duodenal ferroportin: transcriptional activation of its gene via HIF2α (which is negatively regulated by IRP1), translational repression of its mRNA via IRPs, and stabilization of the protein via downregulation of hepcidin. The net result is induction of ferroportin, which is considered a physiologic response to increase iron supply to the bloodstream (McKie et al., 2000). It has been proposed that the IRP blockade can be bypassed via enriched expression of an alternatively spliced non-IRE containing ferroportin transcript (Zhang et al., 2009). Nevertheless, in other reports the canonical IRE-containing ferroportin mRNA was found to be predominant in the duodenum of rats and mice (Darshan et al., 2011; Galy et al., 2013). Another possibility is that protein stabilization due to suppression of hepcidin is dominant.

Likewise, iron deficiency elicits multiple and antagonistic signals on duodenal DMT1: transcriptional activation of its gene via HIF2α (which is negatively regulated by IRP1), possible stabilization of its IRE-containing transcripts by IRPs, and possible stabilization of the protein via downregulation of hepcidin. Again, the net result is induction of DMT1 (Gunshin et al., 1997), which serves to increase dietary iron absorption from the intestinal lumen. Four different DMT1 mRNA isoforms are generated by the combination of alternative promoter usage and alternative splicing, and two of them harbor an IRE (Hubert and Hentze, 2002). Interestingly, the IRE-containing DMT1 mRNAs predominate in the duodenum and their expression is induced in iron deficiency (Canonne-Hergaux et al., 1999; Hubert and Hentze, 2002). Their transcription is selectively activated by HIF2α (Mastrogiannaki et al., 2009; Shah et al., 2009).

Experiments with mice bearing global or enterocyte-specific Irp deficiencies provided insights on the role of the IRE/IRP system in dietary iron absorption. Irp1^−/−^ mice express high levels of duodenal ferroportin and IRE-Dmt1 mRNAs, most likely as a result of de-repression of Hif2α mRNA translation and enhanced Hif2α transcriptional activity in this tissue (Anderson et al., 2013). This interpretation is consistent with the high expression of further Hif2α target genes. Ferroportin and Dmt1 expression was not altered in duodena of Irp2^−/−^ mice, which exhibit iron overload and express high levels of ferritin (Galy et al., 2005b). High levels of duodenal ferritin were also documented in mice with enterocyte-specific disruption of Irp2 (Ferring-Appel et al., 2009). On the other hand, duodenal ferritin was not suppressed in mice expressing the constitutive IRP1 transgene (Casarrubea et al., 2013), in agreement with previous cell culture experiments (Wang and Pantopoulos, 2002), possibly due to alternative ferritin mRNA translation via internal initiation (Daba et al., 2012). Thus, IRP2 alone appears to function as an important regulator of ferritin but not ferroportin and DMT1 in the duodenum.

Mouse pups with enterocyte-specific deletion of both Irp1 and Irp2 are viable at birth but die within 4 weeks due to malabsorption and dehydration, associated with abnormalities in intestinal architecture (Galy et al., 2008). Their enterocytes manifested highly increased expression of ferritin and ferroportin, and reduced levels of Tfr1 and Dmt1. The downregulation of ferritin and ferroportin was not associated with alterations in their mRNA levels, reinforcing the role of IRPs in the regulation of duodenal ferritin synthesis, but also demonstrating a prominent function of the IRE/IRP system in translational control of duodenal ferroportin mRNA. Expression of the major IRE-Dmt1 mRNA isoform was slightly reduced in the mutant mice, providing first *in vivo* evidence for a role of IRPs in the control of IRE-DMT1 mRNA stability.

**Figure 7:**
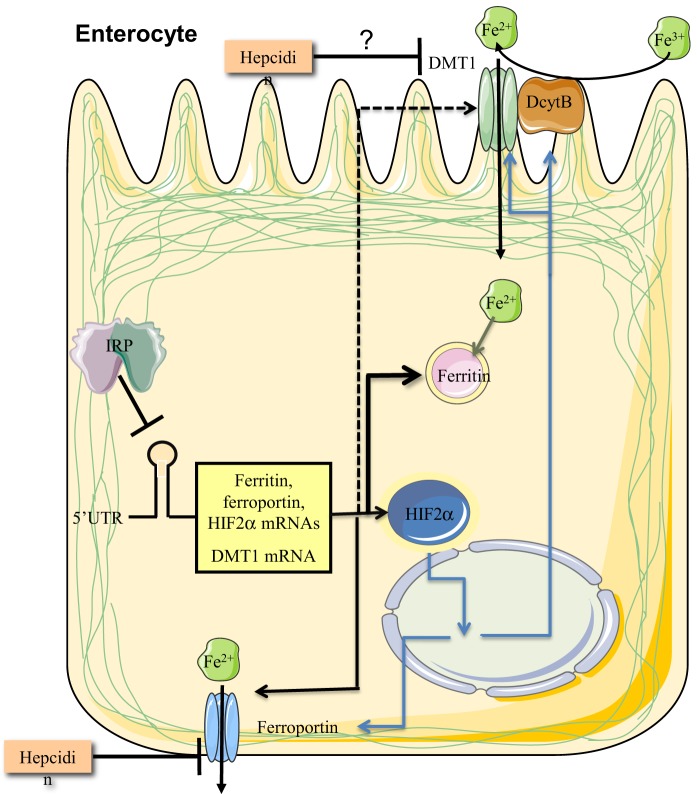
**IRPs control dietary iron absorption by limiting ferritin synthesis in duodenal enterocytes**. Regulated expression of ferritin is essential to prevent a “mucosal block” following iron intake from the intestinal lumen. In addition, IRPs control the expression of ferroportin and DMT1 mRNAs, the latter only in the period after birth and before weaning. Finally, IRP1 controls the expression of HIF2α mRNA, which in turn transcriptionally induces, among other targets, the iron transporters ferroportin and DMT1, and the ferrireductase Dcytb. Ferroportin and possibly also DMT1 are negatively regulated by hepcidin at the level of protein stability.

Adult mice with enterocyte-specific ablation of both Irp1 and Irp2 were generated by using a tamoxifen-inducible Cre-deleter strain under the control of the villin promoter (Galy et al., 2013). These animals are viable and the ablation of Irps does not significantly alter intestinal architecture. As expected, the expression of ferritin and ferroportin was very high, while Tfr1 levels were low. Dmt1 was also upregulated, in contrast to the data obtained in pups with enterocyte-specific deletion of both Irps (Galy et al., 2008). The induction of Dmt1 was associated with an increase in steady-state levels of the IRE-Dmt1 mRNA isoforms, which was attributed to transcriptional stimulation by Hif2α. Together, the above data suggest that Irps regulate the IRE-Dmt1 mRNA only in newborn mice but not in adult animals. This is consistent with the notion that duodenal iron absorption in suckling pups is enhanced to satisfy metabolic needs of the rapidly growing organism, and is not subjected to negative regulation by hepcidin (Darshan et al., 2011). Stabilization of the IRE-Dmt1 mRNA isoforms by Irps could contribute to the increased iron absorption capacity before weaning. Surprisingly, adult mice with enterocyte-specific ablation of both Irps exhibited impaired iron absorption, in spite of the profound induction of the apical and basolateral iron transporters. This was attributed to a “mucosal block” imposed by the overexpression of ferritin, which stores internalized iron and prevents its delivery to the bloodstream. Therefore, alleviation of the “mucosal block” by limiting the expression of ferritin emerges as a crucial function of IRPs, possibly with a major contribution of IRP2, in enterocytes (Figure 7).

## The IRE/IRP system in systemic iron metabolism

Systemic iron metabolism is regulated by hepcidin, a peptide hormone synthesized by liver hepatocytes, which targets ferroportin in duodenal enterocytes, tissue macrophages and other iron-exporting cells (Ganz, 2013). The IRE/IRP system appears to intersect with the hepcidin ferroportin axis. Young Irp1^−/−^ mice exhibit a marked suppression of hepcidin mRNA, accompanied by accumulation of ferroportin in splenic macrophages (Wilkinson and Pantopoulos, 2013). This response is caused by the increased erythropoietic activity of these animals due to induction of Hif2α and Epo, which is an established and potent inhibitor of hepcidin expression (Ganz, 2013). Irp2^−/−^ mice were found to have physiological hepcidin mRNA levels, in spite of hepatic iron overload (Cooperman et al., 2005; Galy et al., 2005b). However, at the age of 4–6 weeks, these animals showed a significant induction of hepcidin mRNA expression (Wilkinson and Pantopoulos, 2013). Conceivably, these seemingly discrepant findings are related to the antagonistic signals of erythropoietic drive and iron overload, which act as negative or positive regulators of hepcidin, respectively (Ganz, 2013). These stimuli do not operate in a strictly hierarchical manner, and their dominance depends on signal strength (Huang et al., 2009). Hepatic iron overload may be dominant in young animals, and later neutralized by the erythropoietic drive. Nevertheless, 8–10-week-old liver-specific Irp2^−/−^ mice, which develop hepatic iron overload without microcytic anemia, exhibit physiological hepcidin expression (Ferring-Appel et al., 2009). This may indicate that Irp2 expression is required for iron-dependent hepcidin induction in mice older than 4–6 weeks, but the underlying mechanism is unclear.

The liver is a major site for excessive iron storage within ferritin, but also the central regulator of systemic iron balance via hepcidin (Meynard et al., 2014). The hepatic iron overload phenotype that was observed in mice with global Irp2 disruption persisted in liver-specific Irp2^−/−^ counterparts, which is indicative of a cell autonomous function (Ferring-Appel et al., 2009). Liver-specific disruption of both Irp1 and Irp2 resulted in early lethality within 1–2 weeks after birth due to liver failure (Galy et al., 2010). This was associated with mitochondrial iron deficiency, as well as histological and functional defects in this organelle. Thus, the IRE/IRP system is essential for liver function. Along similar lines, unregulated overexpression of endogenous Irp2 in liver-specific Fbxl5^−/−^ mice impaired hepatic and systemic iron homeostasis (Moroishi et al., 2011). The animals developed hepatic iron overload and steatohepatitis, and exhibited inappropriately low hepcidin mRNA expression. Moreover, they succumbed after feeding with an iron-enriched diet due to lethal liver failure.

## The IRE/IRP system in cancer

Considering that HIF2α may function either as a tumor promoter or suppressor (Keith et al., 2012), the regulation of HIF2α mRNA translation by IRP1 provides a link between the IRE/IRP system and cancer biology. Other links were previously provided by tumor xenograft experiments. Thus, overexpression of IRP1 or a constitutive IRP1 mutant in human H1299 lung cancer cells impaired tumor xenograft growth in nude mice (Chen et al., 2007). Contrary, the overexpression of IRP2 elicited the opposite phenotype, which required the presence of an IRP2-specific 73 amino acids domain (Maffettone et al., 2010). IRE-containing mRNAs were not differentially expressed in IRP1- or IRP2-overexpressing xenografts, which exhibited distinct gene expression profiles (Maffettone et al., 2010). These data raise the possibility for a role of IRPs in modulating cancer growth independently of their IRE-binding activities, which remains to be further explored. Nevertheless, IRP2 was recently shown to be overexpressed in breast cancer cells and to promote tumor growth by modulating iron metabolism (Wang et al., 2014). This finding is consistent with reprogramming of iron metabolism in cancer cells (Torti and Torti, 2013). Notably, in earlier experiments IRP2 was reported to be transcriptionally induced by the proto-oncogene c-myc and to promote cell transformation by suppressing ferritin and by regulating cellular iron availability (Wu et al., 1999). On the other hand, the tumor suppressor p53 upregulated ferritin by reducing IRP activities (Zhang et al., 2008). Development of cancer models with Irp1^−/−^ and Irp2^−/−^ mice is expected to better define the roles of IRPs in cancer and to elucidate the molecular basis underlying the association of IRP2 (*IREB2*) genomic locus with susceptibility to lung cancer (Hansen et al., 2010; Cho et al., 2012; Fehringer et al., 2012).

## Conclusions

The discovery of the IRE/IRP regulatory system provided a framework to understand the coordinate regulation of cellular iron uptake via TfR1, and storage within ferritin. Misregulation of L-ferritin expression due to “loss of function” mutations in the IRE of its mRNA is clinically relevant and underlies the molecular basis of the hyperferritinemia-caratact syndrome. The expansion of IRE-containing mRNAs raised the possibility that IRPs may control further biochemical pathways. This was firmly established by the analysis of mouse models with global or tissue-specific Irp1 and/or Irp2 deficiency. The animal studies highlighted a key role of the IRE/IRP system in regulation of erythropoiesis, dietary iron absorption, hepatic iron metabolism and body iron homeostasis via crosstalk with the hepcidin/ferroportin axis. These findings may be highly relevant to human medical conditions.

### Conflict of interest statement

The authors declare that the research was conducted in the absence of any commercial or financial relationships that could be construed as a potential conflict of interest.
